# Physicochemical parameter based estimation of discarding points for frying oil using data interpolation and principal component transformation

**DOI:** 10.1002/fsn3.1524

**Published:** 2020-04-02

**Authors:** Yumei Li, Xianbing Cao, Yanping Cao, Yuxu Feng, Jingjun Ji, Jiuying Xie, Xin Wang

**Affiliations:** ^1^ School of Mathematics and Statistics Beijing Technology and Business University (BTBU) Beijing China; ^2^ Beijing Advanced Innovation Center for Food Nutrition and Human Health Beijing Technology and Business University (BTBU) Beijing China; ^3^ Beijing Laboratory of Food Quality and Safety School of Food and Health Beijing Technology and Business University (BTBU) Beijing China

**Keywords:** acid value, carbonyl value, data interpolation and PCT, discarding points estimation of frying oil, total polar compounds

## Abstract

Data interpolation and principal component transformation (PCT) were used to compute the discarding points of a frying oil by measuring the physicochemical parameters—acid value, carbonyl value, and total polar compounds. Herein, the discarding point refers to the time point (associated with the value of each physicochemical parameter) at which the frying oil should be discarded. First, a primary visual analysis was performed for the obtained data by using line charts. Second, a curve interpolation method was used to compute the discarding points for each parameter and thus determine the discarding points for the frying oil. At 190, 205, and 220°C, the frying oil reached the discarding points at 22.1, 17.7, and 13 hr, respectively. The discarding area was also visualized on the corresponding surfaces for the originally obtained data and the interpolated data to investigate the discarding points. Third, the PCT was conducted for the three parameters at each temperature; the discarding point estimation for the three parameters could be reduced to the estimation from the first principal component (FPC), thereby simplifying this process. At 190, 205, and 220°C, the frying oil reached the discarding points when the FPCs were 10.4524, 6.2881, and −1.7629 at the time points 22.1, 17.7, and 13 hr, respectively. Finally, a verification experiment revealed that the correlation between the results obtained by our interpolation method or PCT and the verified data was higher than 0.98, which demonstrates the effectiveness of our method.

## Graphical abstract

Data interpolation is used to estimate the discarding points for the frying oil. Surfaces and lines are used for frying oil data visual analysis and discarding area displaying. Principal component analysis transformation is used to find a comprehensive indicator for estimating discarding points.

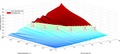

## 1 INTRODUCTION

Fried food is popular among consumers because of its attractive flavor, crispiness and taste, good shape and color, and rich aftertaste. Frying is a widely used means of food processing in the food industry and home cooking. If the frying temperature is significantly high and the frying time is considerably long, many physicochemical changes occur, producing certain harmful substances, which can infiltrate into the food; thus, the food quality and nutritional value is affected, which may harm the human body (Ahmad Tarmizi, Hishamuddin, & Abd Razak, 2019; Lim, Jeong, Oh, & Lee, 2017; Liu, Li, Cheng, & Liu, 2019; Nayak, Dash, Rayaguru, & Krishnan, 2016; Yang, Zhao, & He, 2016; Yu, Cho, & Hwang, 2018). Therefore, it is extremely important to monitor the frying oil quality by considering some physicochemical parameters (Ibrahim, Abd Aziz, Hashim, Jamaludin, & Khaled, 2019; Nayak et al., 2016; Song, Kim, Kim, & Lee, 2017). The most commonly used physicochemical parameters are total polar compounds (TPC; Hammouda et al., 2019; Ibrahim et al., 2019; Li, Li, Wang, Cao, & Liu, 2017; Li et al., 2019; Ma, Zhang, Tuchiya, Miao, & Chen, 2014; Song et al., 2017), acid value (AV; Liu et al., 2019; Ma et al., 2014; Nancy, George, & Ebenezer, 2016; Nayak et al., 2016; Sayyad, 2017; Song et al., 2017), carbonyl value (CV; Shahidi Noghabi, Kaviani, & Niazmdand, 2015; Shyu, Hau, & Hwang, 1998), etc. According to Chinese Hygienic Standard (Ministry of Health of the People's Republic of China, 2004), if the AV, CV, or TPC is ≥5 mg KOH/g, 50 meq/kg, or 27%, respectively, the frying oil should be discarded. Here, the values 5, 50, and 27 can be referred to as the discarding values corresponding to AV, CV, and TPC, respectively. During the actual frying process, if the time point when a parameter exactly reaches the discarding value can be obtained, the discarding time point of the whole frying oil can be determined on the basis of the considered parameters. The discarding time point can be utilized for practical application because the frying oil users can easily estimate the oil change time; additionally, the direct monitoring of the frying oil quality is beneficial for consumers, as well as food safety departments. However, it is difficult to determine the exact time point at which a parameter reaches the corresponding discarding value. Therefore, we can consider the time point at which a parameter value is just greater than or equal to the corresponding discarding value. Here, we call this time point with the corresponding parameter value as the discarding point of that parameter. The whole frying oil should be discarded when one of the studied parameters reaches its discarding point. Therefore, the discarding point of the whole frying oil is the earliest discarding time point obtained for the considered parameters (associated with all the corresponding studied physicochemical parameter values at the time point). Determining the discarding point of a frying oil is not an easy task for scientists or food industry operators (Song et al., 2017); nevertheless, we have attempted to resolve this challenging problem.

The oil's discarding point for a physicochemical parameter can be obtained by continuously measuring or predicting the parameter value on the basis of the obtained data.

However, it is rather tedious and time consuming to measure the physicochemical parameters through chemical analysis (a standard measuring method), which involves chemical reagents and sample preparation steps, and this measurement process is nearly impossible to conduct in real time (Hammouda et al., 2019; Kim, Yu, Kim, Lim, & Hwang, 2018; Shahidi Noghabi et al., 2015). A few rapid measurements, such as piezoelectric (using sensors) and optical property measurements (Ali, Angyal, Weaver, Rader, & Mossoba, 1996; Xu, Zhu, Yu, Huyan, & Wang, 2018), are not suitable to be conducted at high temperatures; moreover, certain parameters cannot be measured because of the limited detection area of the employed equipment. Other rapid measurement methods such as gas chromatography (GC) separation and liquid chromatography (LC) separation (Ali et al., 1996; Feitosa, Boffo, Batista, Velasco, & Silva, 2019; Fritsche et al., 1998; Zribi et al., 2016) also have limitations in measuring the types of parameters because of the different physical and chemical properties. Moreover, these rapid methods cannot directly measure the parameter values; instead, they compute them through conversion between the characteristic values of the piezoelectric frequency, spectra or chromatograms, and the parameter values. Hence, we can surmise that the standard measuring method is more accurate than the other methods because it directly measures the parameters from the fried oil sample (Chen, Chiu, Cheng, Hsu, & Kuo, 2013; Nayak et al., 2016; Wang, Su, Wang, & Nie, 2019; Yang, Zhao, & He, 2016); in addition, the standard measuring method is the most commonly used method, and it can measure many common physicochemical parameters.

On the other hand, because the real‐time measurement of parameters is exceedingly difficult, research is underway to identify alternative time‐saving, labor‐saving, and relatively safer methods for the quick estimation of the changing trends in physicochemical parameters on the basis of the obtained data to monitor the frying oil quality in a better way. Many researchers have predicted the physicochemical parameters through regression or fitting between the physicochemical parameters and frying time, temperature, and other conditions according to the obtained data. The partial least squares regression model has been established between NIR spectra and the AV and TPC (Ma et al., 2014); thus, the AV and TPC can be predicted by observing the NIR spectra, which are measured directly from the hot frying oil. In one study (Li, Wu, Liu, Jin, & Wang, 2015), a regression model between the oil viscosity and temperature was evaluated. In another study (Wang, Su, Wang, & Nie, 2019), a regression model was established between water/oil contents and LF‐NMR parameters. In yet another study (Franklin, Pushpadass, Neethu, Sivaram, & Nath, 2017), a regression analysis was performed between the frying time and the parameters—moisture content, fat content, expansion ratio, apparent density, browning index, and hardness—at 125, 135, and 145°C. Additionally, in various studies (Franklin et al., 2017; Shahidi Noghabi et al., 2015), artificial neural networks (ANN) have been used to predict physicochemical parameters. Considering temperature and time as independent variables, and moisture content, fat content, expansion ratio, apparent density, porosity, browning index, and hardness as dependent variables, the ANN has been used to fit the relationship (Franklin et al., 2017). Considering time, temperature, and concentration of antioxidant tert‐butylhydroquinone as independent variables, and peroxide value (PV), CV, and TPC as dependent variables, the ANN has been used to fit the relationship (Shahidi Noghabi et al., 2015). All of the abovementioned studies only predict the parameter value and do not give a time estimate for a particular parameter to reach the discarding point in their frying environment, except for the following studies reported by Ravelli, Matsuoka, Modesta, and Vieira (2010) and Song Kim Kim and Lee (2017), wherein Ravelli et al. (2010) mainly discussed whether trained panelists could identify the deteriorated frying oil with the same effect as the sensory evaluation does. In their experiments, potato chip portions (400–550 g) were fried in 3.5 L of soybean oil; the frying time of each portion ranged from 8 to 15 min; and fresh oil was added as makeup oil after each frying instance to maintain the original amount of oil in the fryer. The selected and trained panelists gave the conclusion that the disposal time of the soybean oil used for the deep frying of potato chips at a maximum temperature of 180°C is 4 hr by identifying the frying oil on the basis of color, aroma, viscosity, and flavor. Their conclusion was consistent with the partial physicochemical parameters evaluated via applied sensory analysis. Song et al. (2017) believe that determining the discarding points of the used frying oils is not an easy task for scientists or food industry operators. In their experiment, the frying pot was filled with 3.5 kg of fresh soybean oil and heated to 180°C for 170 hr; then, chicken frying was performed 130 cycles. They studied the changes in certain physicochemical parameters during the oil‐heating and frying cycles. They concluded that the TPC values ≥24% were obtained after 109 hr of heating the oil and after 100 cycles for the oil used to fry chicken, and 24% of the TPC was the discarding criterion in a few countries.

From the above discussion, first, the discarding point estimation for a frying oil is quite difficult, whether by continuous measurement or by prediction on the basis of the obtained data. Most of the literature studies only predict the investigated physicochemical parameters for the frying oil and do not explicitly determine the discarding time points for the parameters or for the whole frying oil. Second, from the two studies (Ravelli et al., 2010; Song, Kim, Kim, & Lee, 2017) that reported the discarding time points, different discarding time points can be obtained by varying the operating environments (e.g., oil amount, temperature, types of frying food, or physicochemical parameters). If certain standard and specific frying environments and operating processes could be developed, the quality monitoring of the frying oil would become quite convenient, and the estimation of the discarding point would become quick. Before formulating a specification, we continue to estimate the discarding points in various environments, and this is of great significance. Therefore, we measured relevant data in large intervals of time points via the standard measuring method, which is relatively accurate, and then predicted the physicochemical parameter values in certain intervals of time points to provide relatively accurate discarding points and monitor the frying oil quality. Moreover, a comprehensive study of several physicochemical parameters revealed that the discarding time points for multiple parameters at the same temperature were not consistent with each other. Therefore, finding a comprehensive indicator based on multiple parameters to predict the discarding points for the whole frying oil is also extremely important. Herein, PCT was used for finding the comprehensive indicator.

## 2 MATERIALS AND METHODS

### 2.1 Materials and instruments

Soybean oil was purchased from Zhongliang Eastocean Oils & Grains Industries (Zhangjiagang) Company, Limited. Quick‐frozen potato chips were supplied by Shanghai Sheng Fang Industrial Company, Limited. Sodium hydroxide, ether (absolute), isopropyl alcohol, ethanol, trichloroacetic acid, benzene, potassium hydroxide, and petroleum ether were obtained from Beijing Chemical Works. Phenolphthalein was provided by Tianjin Beichen Fangzheng Reagent Factory. 2,4‐Dinitrophenylhydrazine was obtained from Tianjin Ruijin Chemical Company, Limited.

HH‐S digital display constant temperature oil bath was supplied by Changzhou Rong Hua Instrument Manufacturing Company, Limited. HH‐ZK1 constant temperature control was obtained from Gongyi Yuhua Instrument Liability Company, Limited. The spherical condensation tube and glass dryer were supplied by Beijing Huabo Station Biological Analysis Technology Company, Limited. UV‐9000S double beam UV–vis spectrophotometer was obtained from Shanghai Yuanshan Instrument Company, Limited. The YZF vacuum dryer was purchased from Shanghai Yao Instrument Equipment Factory.

### 2.2 Experimental procedure

The temperatures were set to 160 ± 2, 175 ± 2, 190 ± 2, 205 ± 2, and 220 ± 2°C. The experiments involved setting the oil bath at each of the aforementioned temperatures; 15 L of the soybean oil was poured into the constant temperature oil bath, continuously frying every day for 31 hr. In the entire frying process, the oil temperature was maintained constant, and six batches of potato chips (each batch was 100–200 g) were fried in 1 hr. Each batch of potato chips was fried for 3 min, and then, the fried oil was kept at the same temperature for 7 min to maintain the frying conditions stable. After the oil was heated to the specified temperature, the first 150 ml of the oil sample was taken out, and the corresponding time point was denoted as the 0th hour. Next, the first six batches of potato chips were fried; then, another 150 ml of the oil sample was taken out, and the time was denoted as the 1st hour. These were repeated until the 30th hour was denoted. Totally, 31 oil samples were obtained. Additionally, no new oil was added throughout the frying process as the initial amount of oil that was poured into was sufficient. Each oil sample was cooled to ambient temperature, filtered to remove the solid residues, and then stored in a sample bottle at −20°C for measuring the corresponding physicochemical parameters by using a chemical method. From the 0th to 30th hour, at each temperature, 31 oil samples were taken out for the measurement of each parameter, and 155 oil samples were taken out altogether at five temperatures for measuring the three parameters.

The AV and CV measurements were performed according to the measuring method of GB/T 5009.37‐2003 “Method for Analysis of Hygienic Standard of Edible Oils” (Ministry of Health of the People's Republic of China, 2004).

The TPC measurement was performed according to the measuring method of GB/T 5009.202‐2016 “Determination of polar compounds in edible oils used in frying food” (Ministry of Health of the People's Republic of China, 2016).

### 2.3 Methods

#### 2.3.1 Line chart visualization of obtained data

The obtained data were analyzed in both cases through line charts. One type of line charts shows the changes in one parameter at five temperatures. Another type of line charts shows the changes in the three parameters at each temperature.

#### 2.3.2 Data interpolation for discarding point estimation

**Figure 1:**
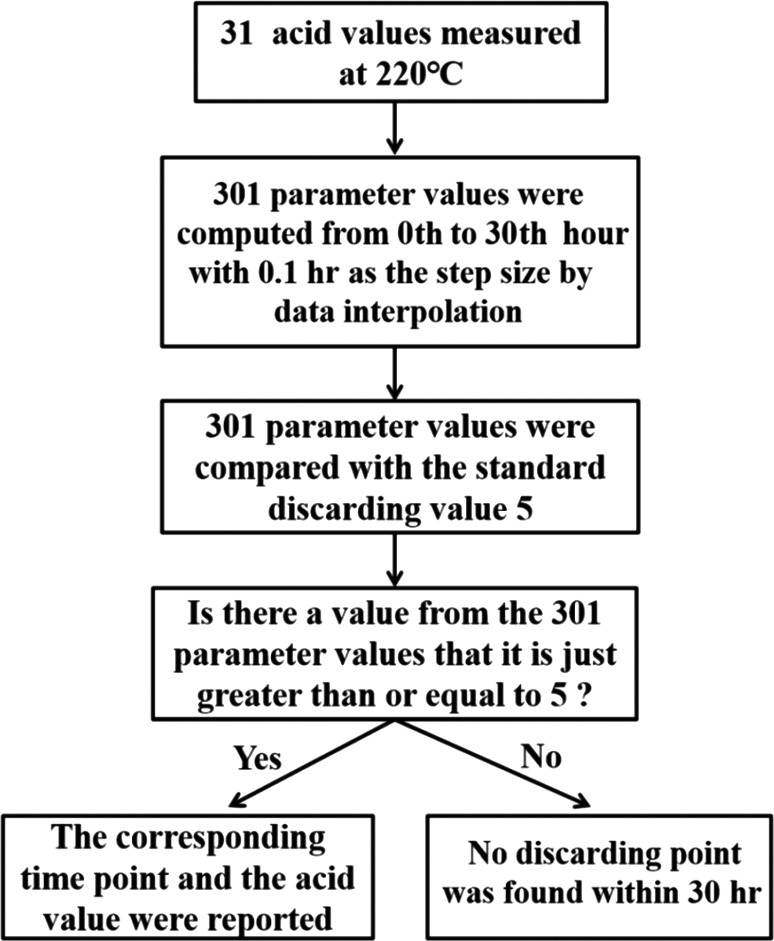
The flowchart of finding discarding point for acid value at 220°C by data interpolation

According to Chinese hygienic standard (Ministry of Health of the People's Republic of China, 2004), we believe that the frying oil should be discarded when the three parameters are ≥5, 50, or 27 (we call these as standard discarding values). At each temperature, we determined the discarding time point and the corresponding parameter value via data interpolation on the basis of the obtained data. With the obtained data as the interpolation nodes and the corresponding 301 time points as the interpolation function nodes, 301 interpolated parameter values were computed through data interpolation in MATLAB, where the 301 time points were obtained from 0th to 30th hour with 0.1 hr as the step size. We compared the 301 interpolated parameter values with the corresponding standard discarding values; if the parameter value was greater than or equal to the standard discarding value, we considered the time point and the corresponding parameter value as the discarding point. If all 301 parameter values were less than the standard discarding values, we concluded that no discarding point was found within 30 hr. Taking the AV at 220°C as an example, we present the discarding point estimation process through data interpolation in Figure 1.

#### 2.3.3 Surface visualization of the parameters and the corresponding discarding area

On the basis of the originally obtained data, the interpolated data were computed at smaller time points from 0th to 30th hour (with 0.1 hr incremental step) and at low‐temperature points from 160 to 220°C (with 1°C as the incremental step) by using MATLAB. Then, two surfaces of the originally obtained data and the interpolated data were drawn. Moreover, we presented the discarding area on the surface with red color and also labeled several discarding points on the surface to see the distribution of the discarding points.

#### 2.3.4 PCT for discarding point estimation

At each temperature, the PCT was carried out on the obtained data for the three parameters by using MATLAB. The contribution rates of the FPC were exceedingly high, and this indicates that the FPC could almost represent all three parameters. Therefore, the discarding points could be estimated in terms of the FPC. We also obtained the transformation expression between the FPC and the three parameters to determine the discarding values from the new parameter values.

## 3 RESULTS AND DISCUSSION

### 3.1 Line charts for each parameter at different temperatures

**Figure 2:**
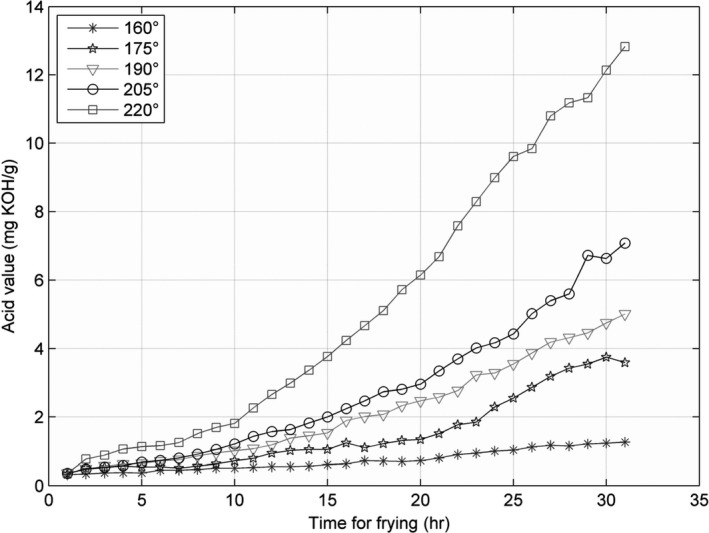
Acid value at five temperatures

**Figure 3:**
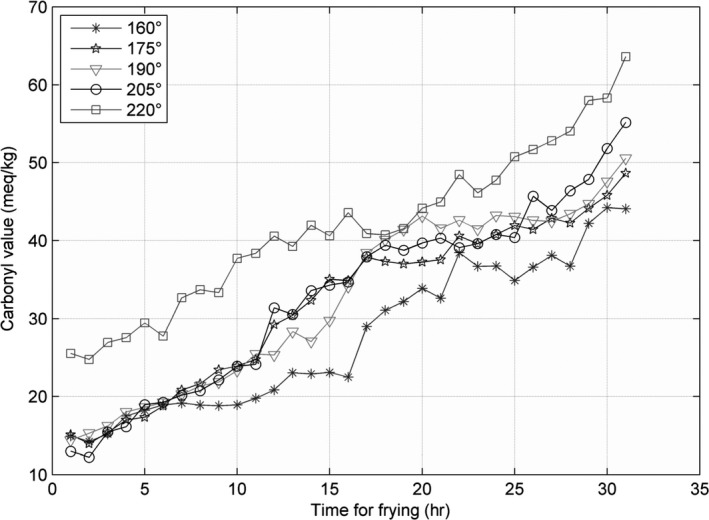
Carbonyl values at five temperatures

**Figure 4:**
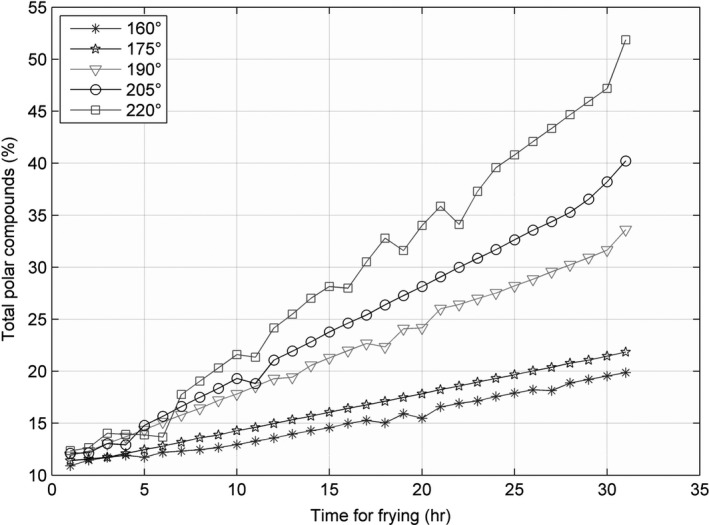
Total polar compounds at five temperatures

The line charts for the three parameters—AV, CV, and TPC—recorded at 160, 175, 190, 205, and 220°C, are shown in Figures 2, 3, 4, and the figures show that the AV, CV, and TPC increase with the increase in time and temperature. The CV grows in an oscillating manner, whereas the AV and TPC grow gradually.

### 3.2 Line charts of different parameters at the same temperature

**Figure 5:**
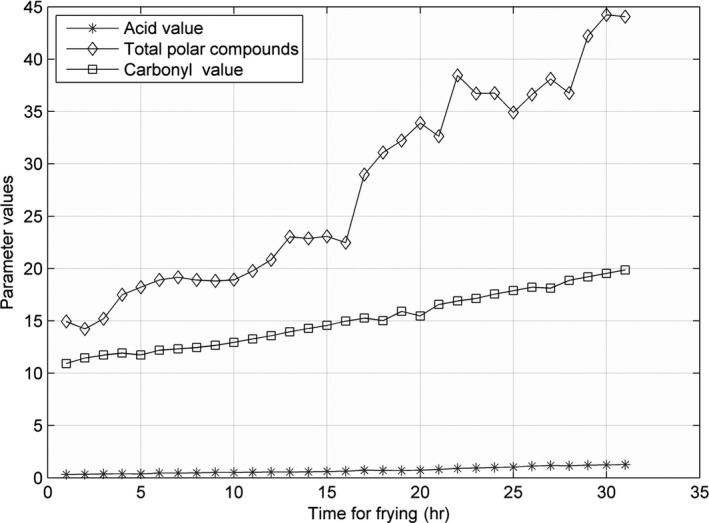
Three parameters at 160°C

**Figure 6:**
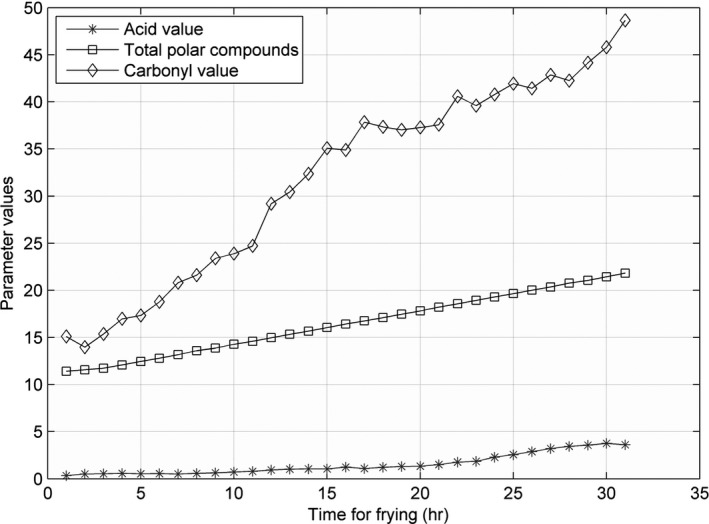
Three parameters at 175°C

**Figure 7:**
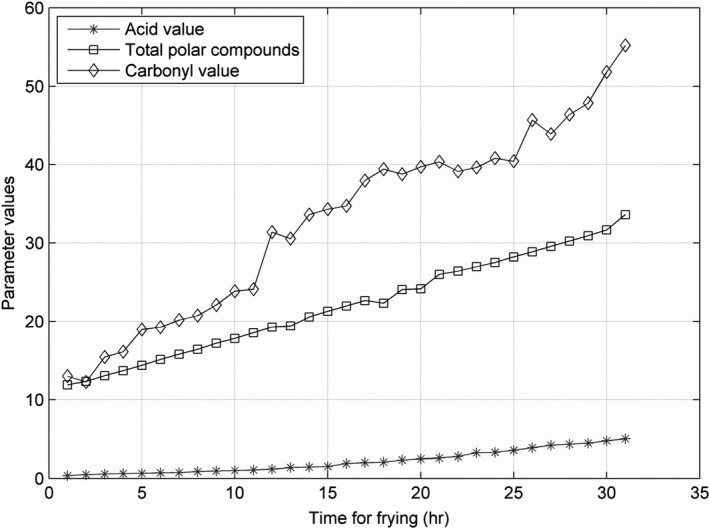
Three parameters at 190°C

**Figure 8:**
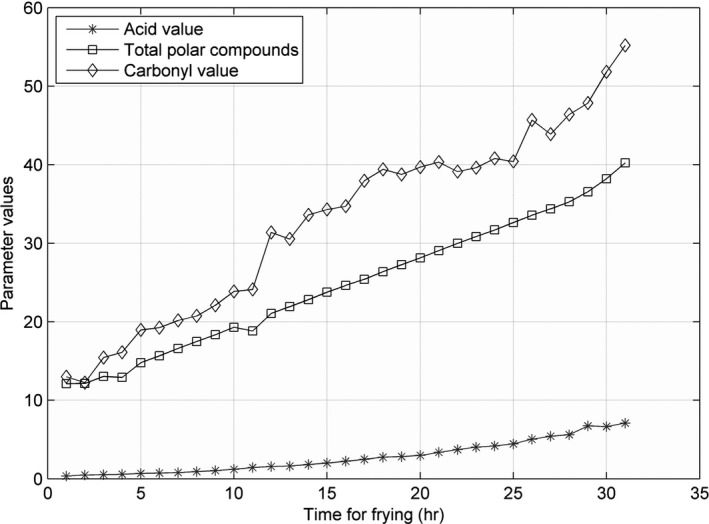
Three parameters at 205°C

**Figure 9:**
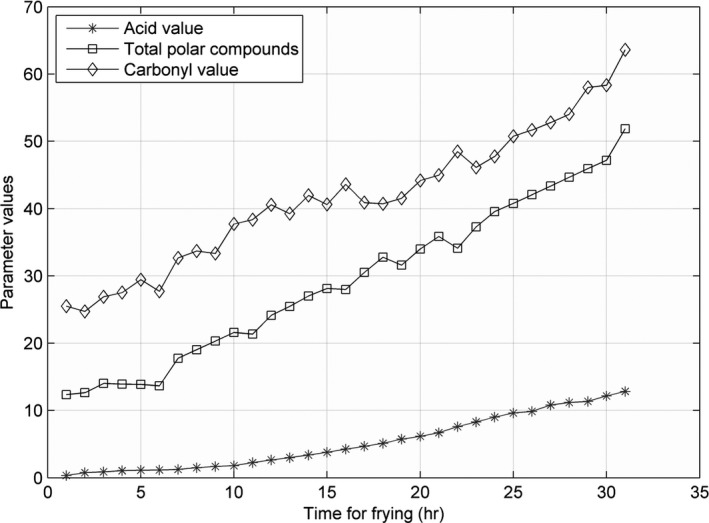
Three parameters at 220°C

The line charts for AV, CV, and TPC, recorded at 160, 175, 190, 205, and 220°C, are shown in Figures 5, 6, 7, 8, 9.

When the temperature was low, the lines were relatively flat for the AV and TPC; thus, the parameters changed slowly. With an increase in temperature, the lines became steeper, and the parameter values sharply increased. However, the CV behaved differently: no matter the temperature is high or low, the change is quite intense. The AV gradually increased with an increase in temperature in the low‐temperature range; however, with further increase in the temperature, the rate of change was slightly higher. The change in TPC was very sharp when the temperature was increased from 190 to 220°C.

### 3.3 Discarding point estimation at different temperatures via data interpolation

All the discarding points found through data interpolation for the three parameters are shown in Tables 1, 2, 3, 4, 5, where the discarding point (29.8 hr 50.0392) in Table 3 indicates that the time point was the 29.8th hour; the corresponding parameter value was 50.0392; and the frying oil should be discarded in terms of the AV. In addition, 30 (No) implies that no discarding point was found within 30 hr. Finally, at each temperature, we considered the lowest discarding time point (among the values obtained from the three parameters) as the final discarding time point for the frying oil, and the corresponding values of the three parameters were also considered at this time. For example, as shown in Table 3, 22.1 hr is the lowest time point, and it is obtained from the interpolation of the CV. Thus, we considered the values of the three parameters (3.2526, 41.4880, and 27.0392) at 22.1 hr from the interpolation results for determining the final discarding point for the frying oil.

**Table 1:** The discarding points at 160°C

160°C	Discarding points reported by data interpolation	Final discarding point of the frying oil
AV	30 (No)	30 (No)
CV	30 (No)	
TPC	30 (No)	

Abbreviations: AV, acid value; CV, carbonyl value; TPC, total polar compounds.

**Table 2:** Discarding points at 175°C

175°C	Discarding points reported by data interpolation	Final discarding point of the frying oil
AV	30 (No)	30 (No)
CV	30 (No)	
TPC	30 (No)	

Abbreviations: AV, acid value; CV, carbonyl value; TPC, total polar compounds.

**Table 3:** Discarding points at 190°C

190°C	Discarding points reported by data interpolation		Final discarding point of the frying oil
AV	30 hr	5.0100	22.1 hr
CV	29.8 hr	50.0392	3.2526 (AV)
TPC	22.1 hr	27.0392	41.4880 (CV)
			27.0392 (TPC)

Abbreviations: AV, acid value; CV, carbonyl value; TPC, total polar compounds.

**Table 4:** Discarding points at 205°C

205°C		Discarding points reported by data interpolation	The final discarding point of the frying oil
AV	25 hr	5.0200	17.7 hr
CV	28.7 hr	50.4058	2.7989 (AV)
TPC	17.7 hr	27.0135	38.8958 (CV)
			27.0135 (TPC)

Abbreviations: AV, acid value; CV, carbonyl value; TPC, total polar compounds.

**Table 5:** Discarding points at 220°C

220°C	Discarding points reported by data interpolation		The final discarding point of the frying oil
AV	16.8 hr	5.0063	13 hr
CV	23.7 hr	50.0198	3.3700 (AV)
TPC	13 hr	27.0100	41.9621 (CV)
			27.0100 (TPC)

Abbreviations: AV, acid value; CV, carbonyl value; TPC, total polar compounds.

For 160 and 175°C, we could not find any discarding points within 30 hr (Tables 1 and 2). With an increase in temperature, the discarding time points shift to lower values (Tables 3, 4, 5); then, the discarding time points for the CV were greater than those for the other two parameters at 205 and 220°C, and this shows that the CV increased relatively slowly when the temperature was increased. However, the TPC reached the discarding point first. This implies that at high temperatures, the TPC increased faster than the other two, and therefore, it decides the discarding point for the frying oil; this may be the reason that many rapid measuring devices are only designed to measure the TPC.

### 3.4 Surface visualization of the three parameters and their discarding area

**Figure 10:**
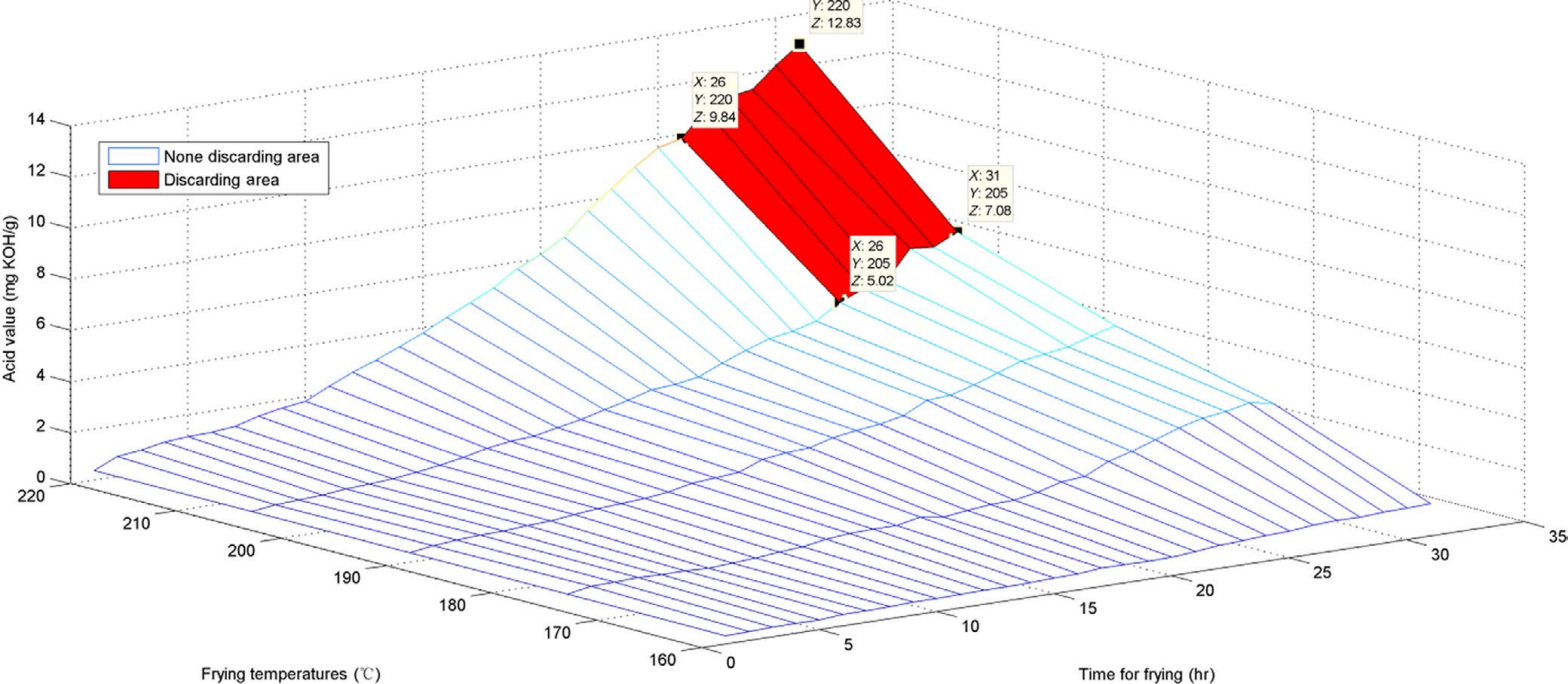
Surface of time–temperature–acid value on the original measured data

**Figure 11:**
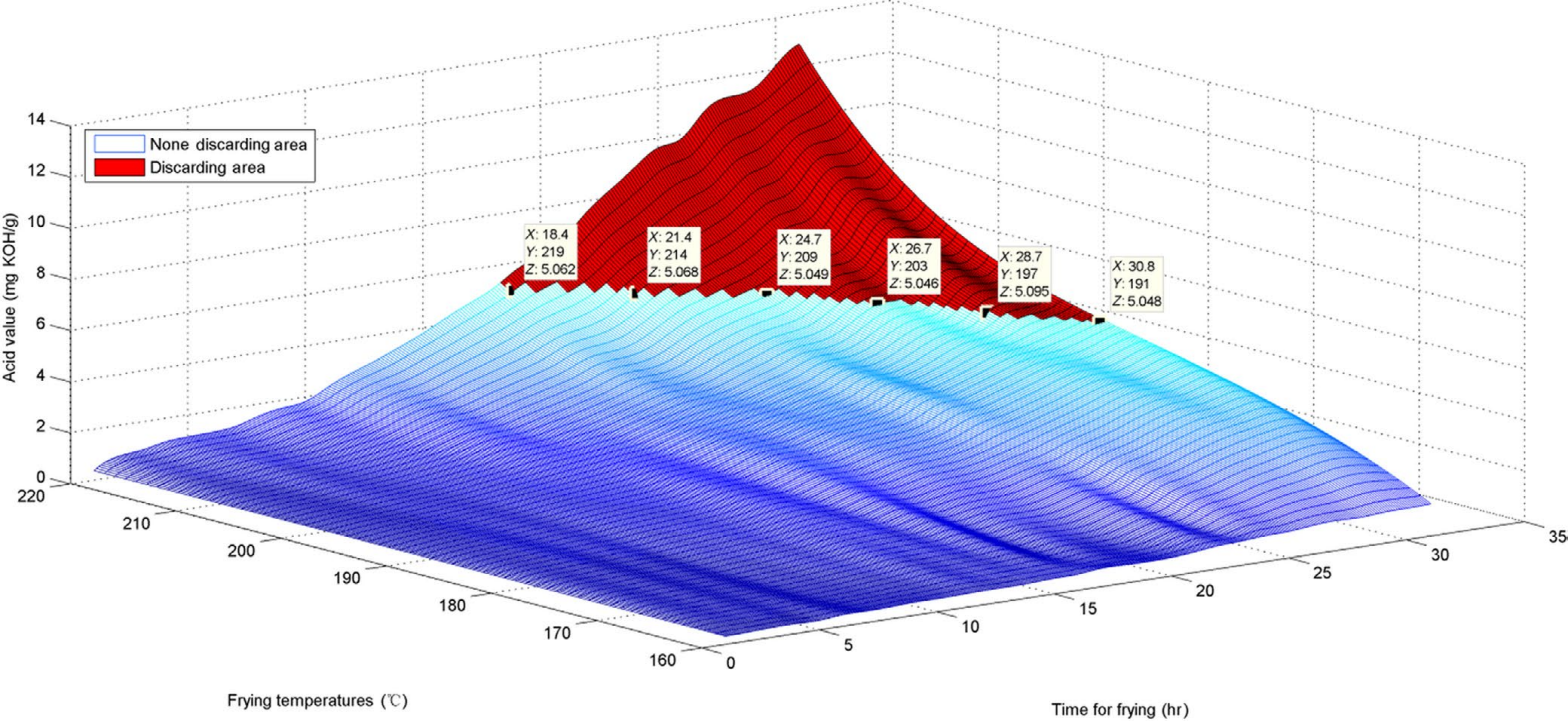
Interpolated surface of time–temperature–acid value

To estimate the AV, the surface for the originally obtained data and the interpolated surface are presented in Figures 10 and 11.

The surface graph in Figure 10 is not quite smooth; nevertheless, the whole surface is relatively uniform. This implies that the AV evenly increased. For the marked discarding points in Figures 10 and 11, *X* represents time, *Y* represents temperature, and *Z* is the interpolated AV. The discarding time decreased with the increase in temperature. Additionally, these marked discarding points were above the horizontal level of *Z* ≥ 5 because the standard discarding value was 5. Moreover, we constructed a red area on the surfaces to indicate the discarding region in which all AVs were ≥5.

**Figure 12:**
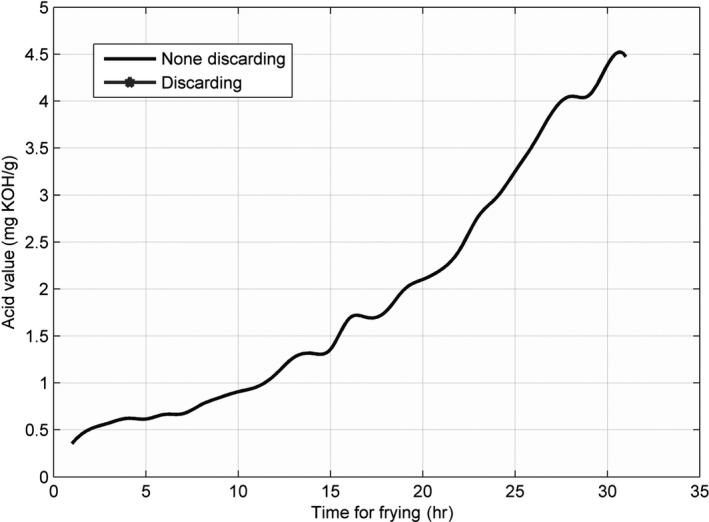
Sliced curve of the acid value at 184°C

**Figure 13:**
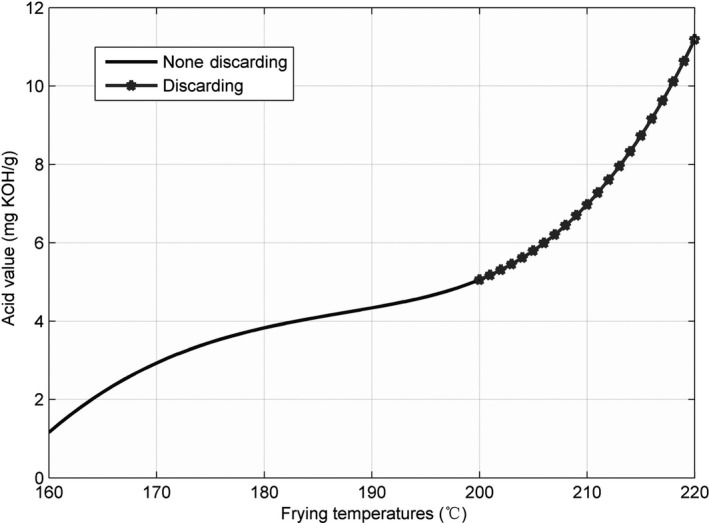
Sliced curve of the acid value at the 28.2 hr

Furthermore, the curves at a particular temperature and at a particular time point were sliced out from the interpolation surface, as shown in Figures 12 and 13.

In fact, from the interpolation surface, we could obtain the sliced curve at any temperature from 160 to 220°C with an interval of 1°C, or we could obtain the sliced curve at any time from the 0th to 30th hour with an interval of 0.1 hr. Therefore, we could observe the change in the parameters at shorter time intervals or lower temperature values. In Figure 12, the last small segment of the line slightly bends down, indicating a decrease in the parameter values; this decrease may be because the last two values at 175°C in the original data showed a downward trend, thereby affecting the interpolation. In Figure 13, the last part of the line implies that the AVs were ≥5, and the oil should be discarded.

**Figure 14:**
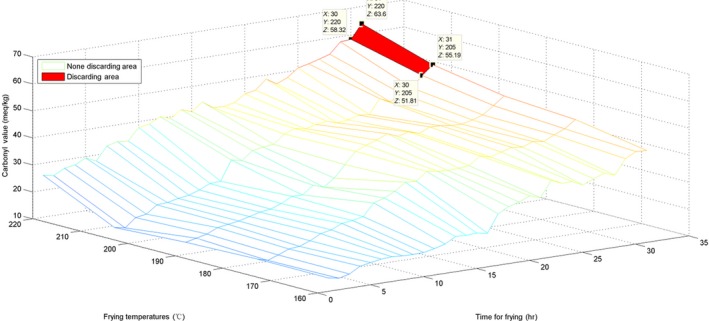
Surface of time–temperature–carbonyl value on the original measured data

**Figure 15:**
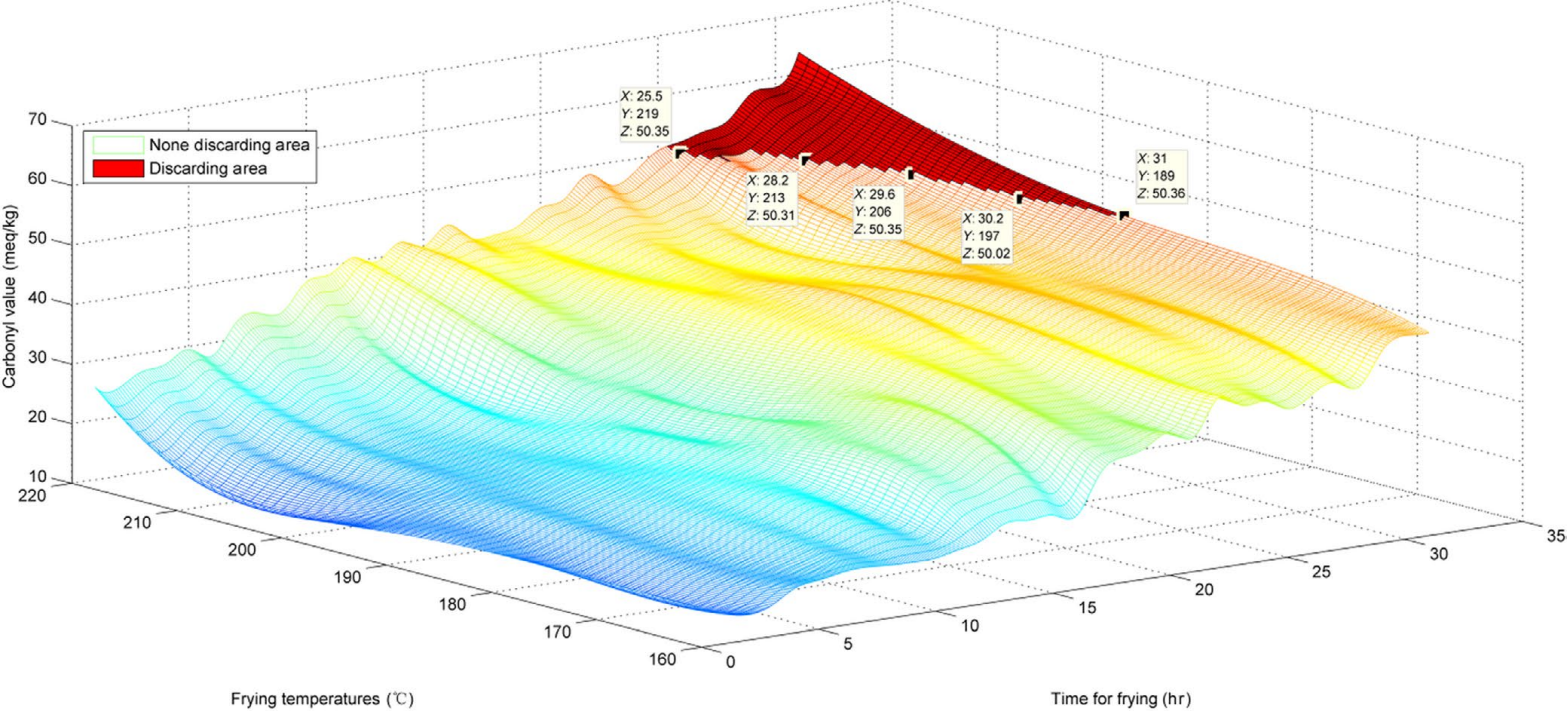
Interpolated surface of time–temperature–carbonyl value

For estimation of the CV, the surface of the originally obtained data and the interpolated surface are presented in Figures 14 and 15.

Figure 14 shows that the surface growth is not uniform; the surface in Figure 15 is also not too uniform and has some irregular pits. This phenomenon also indicates that the CVs at various temperatures and time points were somewhat different from the AVs and the TPC. The standard discarding value is 50, and the *Z* coordinates of the marked points were ≥50. Moreover, with an increase in temperature, the discarding point decreased in value. Similarly, we have labeled the red discarding area on surfaces to indicate the discarding region in which all CVs were ≥50.

**Figure 16:**
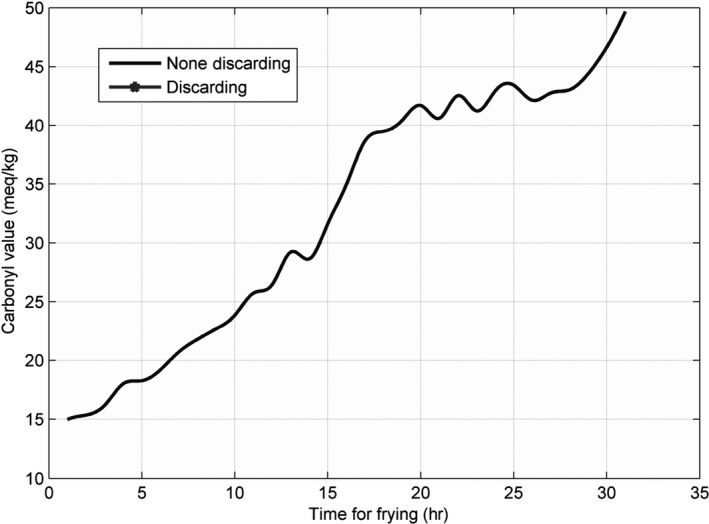
Sliced curve of the carbonyl value at 184°C

**Figure 17:**
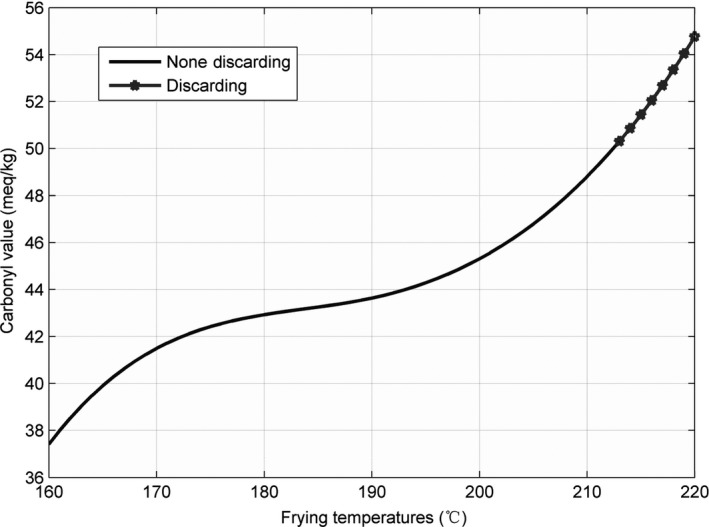
Sliced curve of the carbonyl value at the 28.2 hr

The similarly sliced curves for the CV are shown in Figures 16 and 17.

As shown in Figure 16, at 184°C, the CV increased, accompanied by oscillations. In Figure 17, at the 28.2th hour, the CV rapidly increased at temperatures below 180°C and above 200°C and gradually increased in the temperature range 180–200°C. In Figure 17, the last part of the line indicates that the CVs were ≥50, and the oil should be discarded.

**Figure 18:**
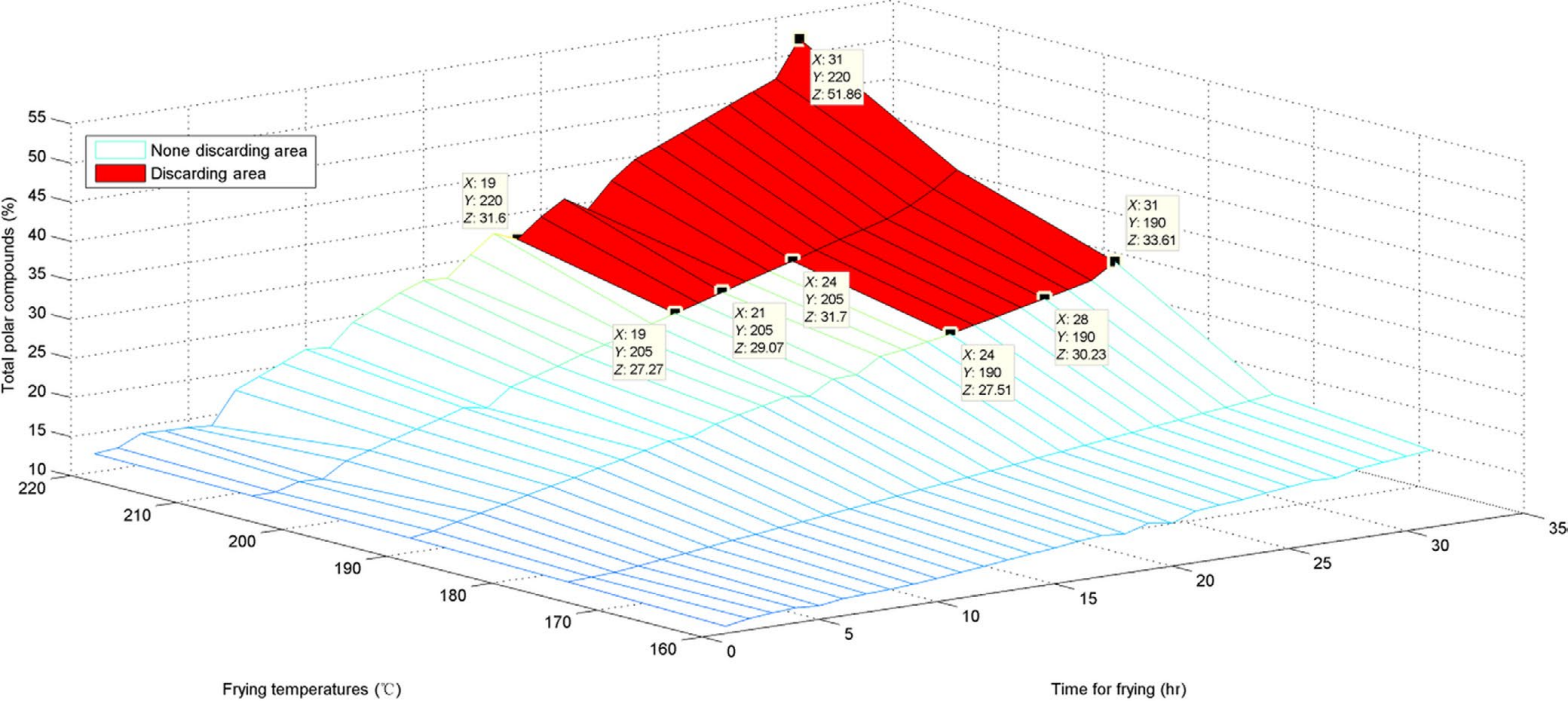
Surface of the original measured data of time–temperature–total polar compounds

**Figure 19:**
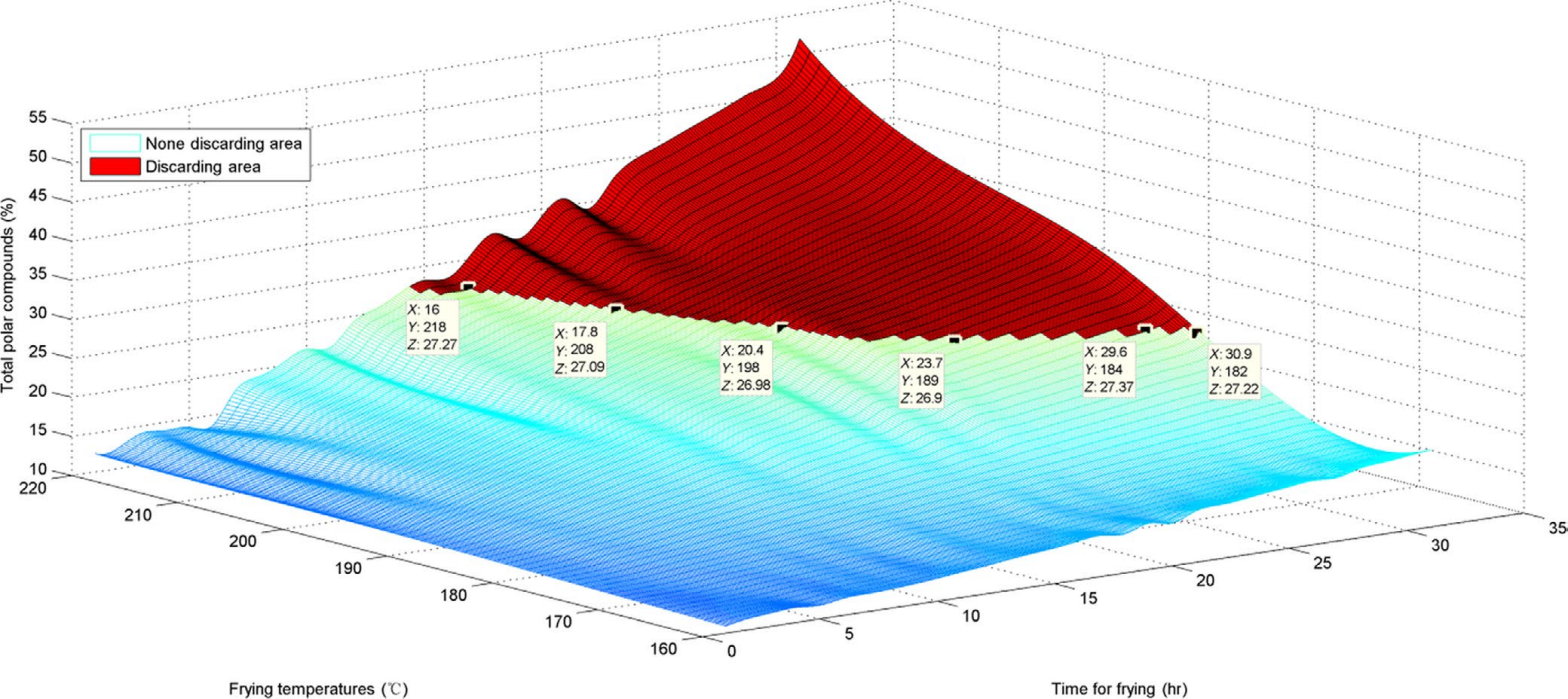
Interpolated surface of time–temperature–total polar compounds

For the TPC, the surface of the originally obtained data and the interpolated surface are presented in Figures 18 and 19.

The surfaces for the TPC were more similar to those for the AVs; only their standard discarding values were different.

**Figure 20:**
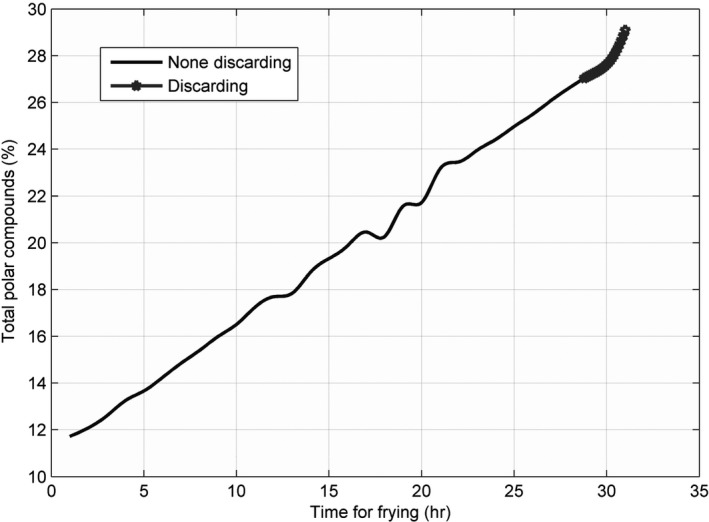
Sliced curve of the total polar compounds at 184°C

**Figure 21:**
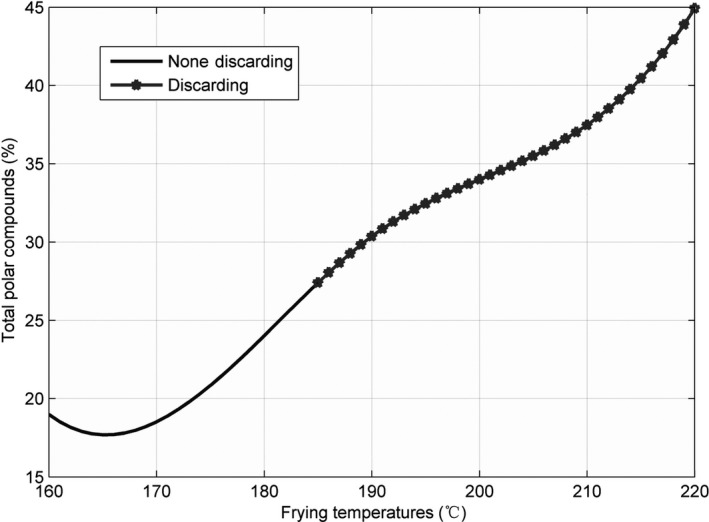
Sliced curve of the total polar compounds at 28.2 hr

The similarly sliced curves for TPC are shown in Figures 20 and 21.

The first few values at 175°C were smaller than those at 160°C in the originally obtained data; therefore, after interpolation, the sliced curve in Figure 21 was concave at approximately 165°C. As shown in the two figures, the values still increased with the increase in temperature over time. The last part of the lines implies that the oil should be discarded.

### 3.5 Discarding point estimation based on PCT

The cumulative contribution rate for the FPC is shown in Table 6. The contribution rate of the FPC was exceedingly high, indicating that the FPC could almost represent all of the three parameters. We selected the FPC to analyze the discarding points and denoted it as *y*. Additionally, we obtained the expression between *y* and the three parameters (AV as *x*_1_, CV as *x*_2_, and TPC as* x*_3_), as presented in Table 7.

**Table 6:** Contribution rate of the FPC

Temperature (°C)	Contribution rate of the FPC
160	0.9962
175	0.9944
190	0.9876
205	0.9910
220	0.9875

Abbreviation: FPC, first principal component.

**Table 7:** Expression between the FPC and the three parameters

Temperature (°C)	Expression
160	$y=0.0293\times x_{1}+0.9629\times x_{2}+0.2638\times x_{3}$
175	$y=0.0846\times x_{1}+0.9538\times x_{2}+0.2884\times x_{3}$
190	$y=0.1045\times x_{1}+0.8721\times x_{2}+0.4780\times x_{3}$
205	$y=0.0196\times x_{1}+0.8233\times x_{2}+0.5673\times x_{3}$
220	$y=0.2435\times x_{1}+0.6379\times x_{2}+0.7306\times x_{3}$

*y*: FPC; *x*_1_: AV,* x*_2_: CV,* x*_3_: TPC.

Abbreviation: AV, acid value; CV, carbonyl value; FPC, first principal component; TPC, total polar compounds.

The coefficients of the CV were relatively larger than those of the other two, and their contribution to the first component was the greatest. The coefficients of the AV were relatively smaller, and their contribution to the first component was the lowest. The coefficients of the TPC were in between the other two, except in the expression at 220°C. These coefficients suggest that the CV was sensitive and that it played a great role in the PCT. When the temperature reached 220°C, the change in the TPC was severe.

Afterward, at a certain temperature, we could choose the three parameter values from the last columns in Tables 3, 4, 5 as* x*_1_,* x*_2_, and* x*_3_; we subtracted the mean values of the corresponding original data in terms of the rule of the PCT and plugged them into the expression given in Table 7; *y* could be computed as the discarding point for the FPC. The computed results are presented in Table 8.

**Table 8:** Discarding points obtained by FPC

Temperature (°C)	Time (hr)	Discarding point of FPC
190	22.1	10.4524
205	17.7	6.2881
220	13	−1.7629

Abbreviation: FPC, first principal component.

For example, at 205°C, we considered the three parameter values (5.6, 46.38552, and 35.28) when the time point was 27.1 hr according to the interpolated data for the three parameters; we subtracted the corresponding mean values of the original data and plugged them into the expression *y* = 0.0196 × *x*_1_ + 0.8233 × *x*_2_ + 0.5673 × *x*_3_. We obtained *y* = 17.4217 > 6.2881. Thus, we concluded that the frying oil should be discarded at the 27.1 hr time point.

## 4 VERIFICATION EXPERIMENT

The three parameters were measured again for verification at the temperatures—190, 205, and 220°C—and the respective time points—22.1, 17.7, and 13 hr. We also considered the interpolated values at the corresponding temperatures and time points for verification. All these data are presented in Table 9; for example, in the third column and second row, 2.2310, 44.9368, 25.5300 implies that the AV was 2.2310, the CV was 44.9368, and the TPC was 25.5300 at 190°C and the 17.7th hour. In addition, according to the last three expressions in Table 7, we could compute the FPC values from the verification data and the interpolated data as in the fourth column and the sixth column in Table 9.

**Table 9:** Verification between new measured verification data and the interpolated data

Temperature (°C)	Time (hr)	Verification data (VD)	FPC from VD	Interpolated data (ID)	FPC from ID
190	22.1	1.5830, 44.3128, 26.3700	12.4214	3.2526, 41.4880, 27.0392	10.4524
205	17.7	2.2310, 44.9368, 25.5300	10.4579	2.7989, 38.8958, 27.0135	6.2881
220	13	2.6291, 50.3472, 24.4200	1.5134	3.3700, 41.9621, 27.0100	−1.7629

Abbreviation: FPC, first principal component.

According to Table 9, the correlation coefficients for the data in each grid of the third, and fifth columns were computed as 0.9989, 0.9899, and 0.9840, respectively. For example, the coefficient for 1.5830, 44.3128, 26.3700 in the third column and 3.2526, 41.4880, 27.0392 in the fifth column was 0.9989. This implies that the interpolation method is quite effective. Table 9 also shows that the correlation coefficient for the fourth column and the sixth column is 0.9852, and thus, the PCT is appropriate and effective. Furthermore, according to the last column in Table 9, the discarding points of the frying oil for the FPC were 10.4524, 6.2881, and −1.7629, and thus, all the transformed FPC values of the verification data 12.4214, 10.4579, 1.5134 were greater than the former, suggesting that all the newly measured three oil samples should be discarded.

## 5 CONCLUSION

This study evaluated the AV, CV, and TPC during the frying process from 0 to 30 hr at 160, 175, 190, 205, and 220°C. The originally obtained data revealed that the CV was more sensitive than the other two. According to the interpolation data, at temperatures below 190°C, no discarding points were found within 30 hr; at 190°C, the oil reached the discarding point at the 22.1th hour with the three parameters—3.2526, 41.4880, and 27.0392; at 205°C, the oil reached the discarding point at the 17.7th hour with the three parameters—2.7989, 38.8958, and 27.0135; at 220°C, the oil reached the discarding point at the 13th hour with the three parameters—3.37, 41.9621, and 27.01. Thus, the TPC took the lead in reaching the discarding points at temperatures higher than 190°C and served as the judging criterion for the discarding points of the frying oil. From the surface visualization and the marked discarding points on the interpolated surface, the discarding points shifted to lower values with increasing temperature, and we also could observe the sensitivity in the CVs from the fluctuation of the surfaces about CV. Finally, we discovered that the FPC could represent almost all the information, and thus, we estimated the discarding points of the frying oil by using the FPC. At 190, 205, and 220°C, the frying oil reached the discarding point when the FPCs were 10.4524, 6.2881, and −1.7629, respectively. Additionally, the verification experiment demonstrated that the data interpolation and PCT were very effective.

## CONFLICT OF INTEREST

The authors declare that they do not have any conflict of interest.

## ETHICAL APPROVAL

This study does not involve any human or animal testing.

## INFORMED CONSENT

Written informed consent was obtained from all participants.
